# Molecular cloning, expression, and in situ hybridization analysis of *MnGPx-3* and *MnGPx-4* from oriental river prawn, *Macrobrachium nipponense*, in response to hypoxia and reoxygenation

**DOI:** 10.1371/journal.pone.0229171

**Published:** 2020-02-21

**Authors:** Lei Xu, Ming Yang, Hongtuo Fu, Shengming Sun, Hui Qiao, Wenyi Zhang, Yongsheng Gong, Sufei Jiang, Yiwei Xiong, Shubo Jin, Yan Wu

**Affiliations:** 1 Wuxi Fisheries College, Nanjing Agricultural University, Wuxi, Jiangsu, People’s Republic of China; 2 Key Laboratory of Freshwater Fisheries and Germplasm Resources Utilization, Ministry of Agriculture, Freshwater Fisheries Research Center, Chinese Academy of Fishery Sciences, Wuxi, Jiangsu, People’s Republic of China; Universite Clermont Auvergne, FRANCE

## Abstract

Glutathione peroxidase (GPx) has been the focus of increased research because of its important role as an antioxidant and in reactive oxygen species (ROS) induced damage repair. Studies on GPxs have relevance with *Macrobrachium nipponense* because it has poor tolerance to hypoxia in *Macrobrachium nipponense*. The two subunits named as *MnGPx-3* and *MnGPx-4* according to the glutathione peroxidase nomenclature system. Both full-length cDNAs were cloned from the hepatopancreas. In this study, we analyzed the expression of two GPxs in *Macrobrachium nipponense* in response to changes in environmental oxygen. Expression levels of *MnGPx-3* and *MnGPx-4* indicated that both have strong responses to hypoxia. In situ hybridization showed that *MnGPx-3* and *MnGPx-4* were located in secretory and storage cells in hepatopancreas. These results suggest that GPx gene is expressed and released by secretory cells and released response to hypoxia. In the gill tissue, however, GPxs are located in blood cells, suggesting that they perform different functions in different tissues or organs. The results of in situ hybridization were consistent with those of quantitative Real-time PCR. This study provides a basis for understanding the oxidative stress response in *M*. *nipponense* under hypoxia.

## Introduction

Dissolved oxygen (DO) has been widely accepted as the main indicator of water quality in the aquaculture of prawns [1]. When organisms are adversely stimulated by the external environment, the balance between oxidation and the antioxidant system is disrupted. Once this balance is broken, it leads to increased production of reactive oxygen species (ROS) and further oxidative stress [2–3]. Previous evidence indicates that ROS are formed by electrons that accumulate during hypoxia [4], and acute reoxygenation after hypoxic treatment can also lead to the formation of ROS [5]. Excess ROS can cause changes in the structure and activity of many active substances, eventually leading to oxidative and metabolic damage [1, 6]. To prevent damage from ROS, cells have developed a set of antioxidant defense systems, including glutathione peroxidase (GPx), superoxide dismutase (SOD), and catalase (CAT), to maintain the redox balance and prevent oxidative damage [7].

Glutathione peroxidase (GPx) plays an important role in restoring ROS-induced damage, protecting DNA, proteins, and lipids from oxidative damage by removing excess ROS [8–10]. GPx has been studied extensively because of its important role in removing excess ROS. Previous studies have shown that the enzyme activity of GPx increases rapidly over a short period in the blood lymphocytes of *Exopalaemon carinicauda* during ammonia nitrogen stress [11]. Furthermore, the enzyme activity of GPx increases and then decreases in the intestinal tract and hepatopancreas of *Litopenaeus vannamei* in response to Aflatoxin B1 (AFB1) [12]. The same trend occurred in the muscles and hepatopancreas of *Litopenaeus vannamei* when exposed to reoxygenation [13]. With these developments in research, the molecular structure of GPx has attracted more and more attention. In mammals, eight GPx coenzymes have been identified according to their subcellular localization, specific functions and structural characteristics [14]. It is worth noting that some GPxs functions have been studied including cytosolic GPx (GPx-1), gastrointestinal GPx (GPx-2), plasma GPx (GPx-3), phospholipid hydroperoxide GPx (GPx-4) and olfactory epithelium GPx (GPx-6) [14–15]. In addition to their function in mammals, GPx also has strong biocatalytic and detoxifying functions in aquatic organisms [16–18]. GPx-1 has been fully studied in *Acrossocheilus fasciatus* [19], and a GPx-4 has been reported in *Penaeus monodon* [20]. However, GPx has not been investigated in *Macrobrachium nipponense*.

As an important commercial prawn species, *M*. *nipponense* is widely distributed in freshwater and low-salinity estuarine regions in China and other Asian countries. Compared with other crustaceans, *M*. *nipponense* has more difficulty adapting to environments with low [21–23]. Therefore, it is important to understand the molecular mechanisms in *M*. *nipponense* in response to hypoxia and reoxygenation in order to promote the sustainable development of prawn aquaculture. In this study, two GPx subunits were cloned and characterized from *M*. *nipponense*. Furthermore tissue expression levels of two GPx subunits under different environments were measured by QPCR. We hypothesize that these two subtypes play different roles in different tissues. We also use in situ hybridization (ISH) to investigate the localization of *MnGPx-3* and *MnGPx-4* mRNA in different tissues.

## Materials and methods

This research was approved by the Institutional Animal Care and Use Ethics Committee of Agriculture Ministry Key Laboratory of Healthy Freshwater Aquaculture, Zhejiang Institute of Freshwater Fisheries.

### Experimental animals and hypoxia environmental settings

In total, 300 healthy adult *M*. *nipponense* were obtained with a wet weight is 3.0±0.5 g. In accordance with previous studies, all prawns were raised in six 500 L tanks at the appropriate parameters in a laboratory with aerated water for a week to adapt to the new environment [24–25].

In the hypoxic experiment, 300 prawns were randomly divided into two groups (normal oxygen and hypoxia), and each group was set with three biological repeats. The oxygen concentration of the normoxic group was maintained at 6.0 ± 0.2 mg O_2_ L^-1^, whereas in the hypoxic group it was maintained at 2.0±0.2 mg O_2_ L^-1^ by bubbling with N_2_ gas [23]. A portable dissolved oxygen meter was used to monitore the change of dissolved oxygen in real time (Mettler Toledo, Switzerland). After the challenge with hypoxia, normal oxygen conditions were restored. The target tissues of six prawns were collected at 0, 12 and 24 h during the hypoxia process and 12 and 24 h of the reoxygenation process after 24h of hypoxia, and frozen immediately in liquid nitrogen. The samples were stored in ultra-low temperature freezer for the next steps (ThermoFisher, USA).

### Cloning of two MnGPxs cDNAs

A partial fragment of opsin cDNA was obtained from transcriptomic cDNA library of the *M*. *nipponense* hepatopancreas (NCBI, SRA051767.2) created in our lab. The isolation of total RNA and the synthesis of first-strand cDNA and 3’-rapid amplification of cDNA ends (RACE) was performed using relevant kits [1]. All primers for these steps are listed in Table 1. The purification of PCR products and sequencing were completed according to instructions.

**Table 1:** Primers used in this study.

Primer	Primer sequence (5’-3’)
*MnGPx-3*-F (ORF)	TACGTGGAGTTTGGATTGTCCTC
*MnGPx-3*-R (ORF)	GTTATGGAAATGCTACCGCTGAC
*MnGPx-4*-F (ORF)	AAAAGCAAACGACGAAGTCACAG
*MnGPx-4*-R (ORF)	ATAGTTCAACAGCAGGAGCAATG
3’ -*MnGPx-3* (3’ RACE out primer)	GCGAGAAGGAACACCCTCTATAC
3’ -*MnGPx-3* (3’ RACE in primer)	TATGTTCCTCCTCCAGCAAGATG
3’ -*MnGPx-4* (3’ RACE out primer)	TGTCGCAAGTAAGTGAGGTTTGA
3’ -*MnGPx-4* (3’ RACE in primer)	TCTAAACAAGGTGGAACTCTGGG
*MnGPx-3*-F (Real-time PCR primer)	TGCTTCATGAGTCTTACGACAAC
*MnGPx-3*-R (Real-time PCR primer)	GTATAGAGGGTGTTCCTTCTCGC
*MnGPx-4*-F (Real-time PCR primer)	ATGGAGAAGGGACTCACCCAATA
*MnGPx-4*-R (Real-time PCR primer)	GAATAGGGTTGGTCTGTGGAGAG
EIF-F (Real-time PCR primer)	CATGGATGTACCTGTGGTGAAAC
EIF-R (Real-time PCR primer)	CTGTCAGCAGAAGGTCCTCATTA

### Bioinformatics analysis and nucleotide sequence

NCBI related online analysis software was used to complete the deduction of amino acid sequence, protein analysis and nucleotide sequence analysis according to the previous method of our laboratory [1]. Multi-sequence alignment and phylogenetic tree construction were completed with DNAMAN 6.0 and MEGA 5.1, respectively.

### Quantitative Real-time PCR (QPCR) analysis of two MnGPx expression

The mRNA expression levels of the two MnGPx subunits following different treatments were measured by QPCR. Synthesis of the cDNAs and subsequent QPCR steps were completed as described previously [1, 26]. According to previous laboratory studies, eukaryotic translation initiation factor 5A (EIF) was chosen as the reference gene of the relative quantification [27–28]. The mRNA expression levels of the two subunits were calculated by using the 2^−ΔΔCT^ method [29].

### In situ hybridization (ISH)

Hepatopancreas and gill samples were collected according to the requirements of the chromogenic in situ hybridization (CISH) technique. Fixed paraffin-embedded sections, standard deparaffinization, sectioning and staining were prepared as previously reported by our laboratory [30]. Slides were evaluated using a light microscope. The following sequences were selected as CISH probes of *MnGPx-3* and *MnGPx-4*, respectively, 5′-CTTGTAGTAGAGGGCGTTTGGATCAGCGAAAGCG-3′ and 5′-CAATGGCAGGAATAGGGTTGGTCTGTGGAGAG-3 ′.

### Statistical analysis

There were biological replicates for all experiments (n = 3). All data were analyzed for ANOVA by selecting the appropriate method using SPSS 20.0 software. According to the previous literature, p ≤ 0.05 was considered to be significant [1, 30].

## Results

### *MnGPx-3* and *MnGPx-4* coding sequences

**Fig 1:**
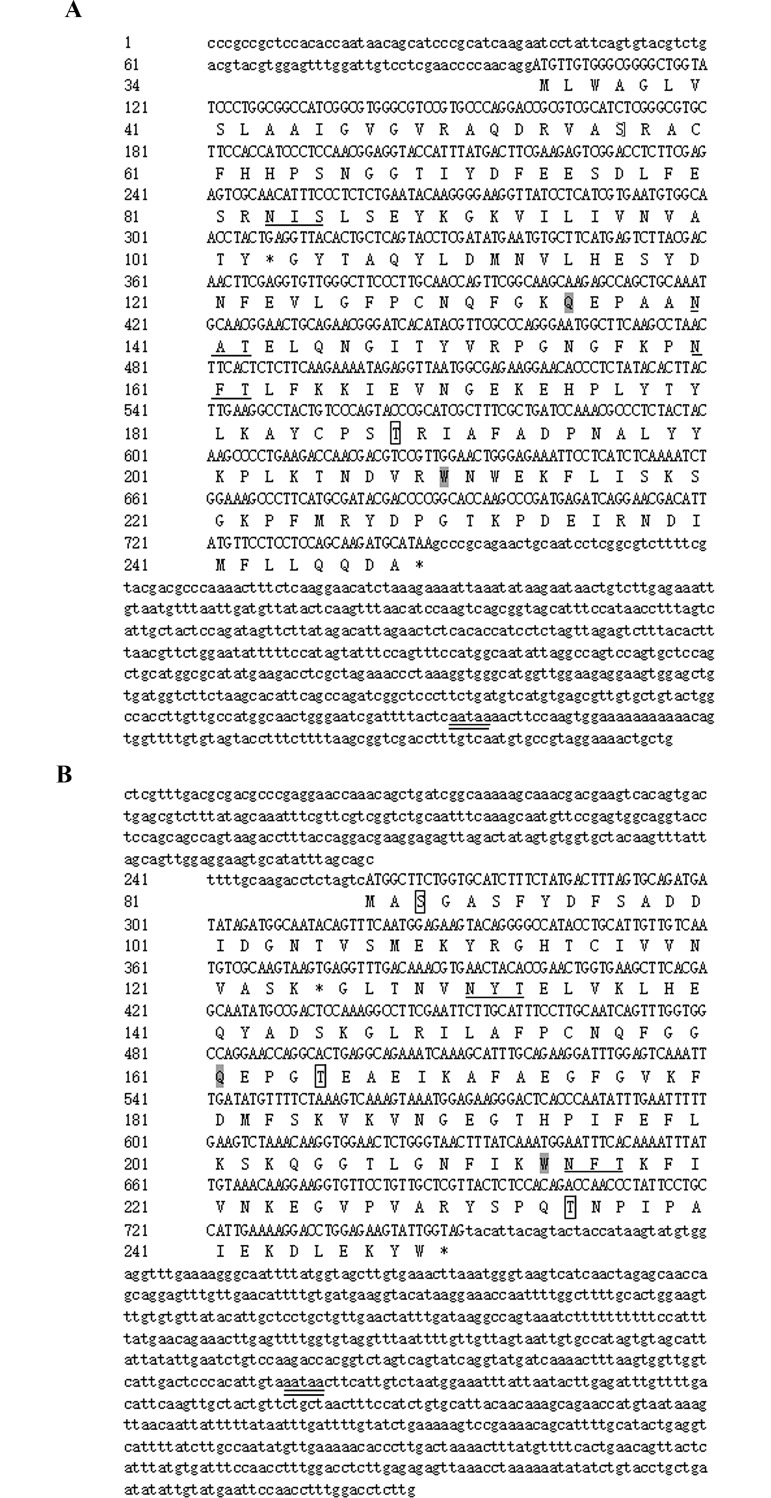
Nucleotide and deduced amino acid sequence of *MnGPx-3*(A) and *MnGPx-4*(B) from the oriental river prawns. Nucleotide and predicted amino acid sequences of *MnGPx-3*(A) and *MnGPx-4*(B) from *M*. *nipponense*. Amino acid sequences are shown as capital letters below the nucleotide sequences. The O-GlcNAc sites, the glycosylation sites, and the active sites are sequentially represented by black rectangles, black underlines, and gray shade. The potential polyadenylation signal (AATAAA) is double underlined.

**Fig 2:**
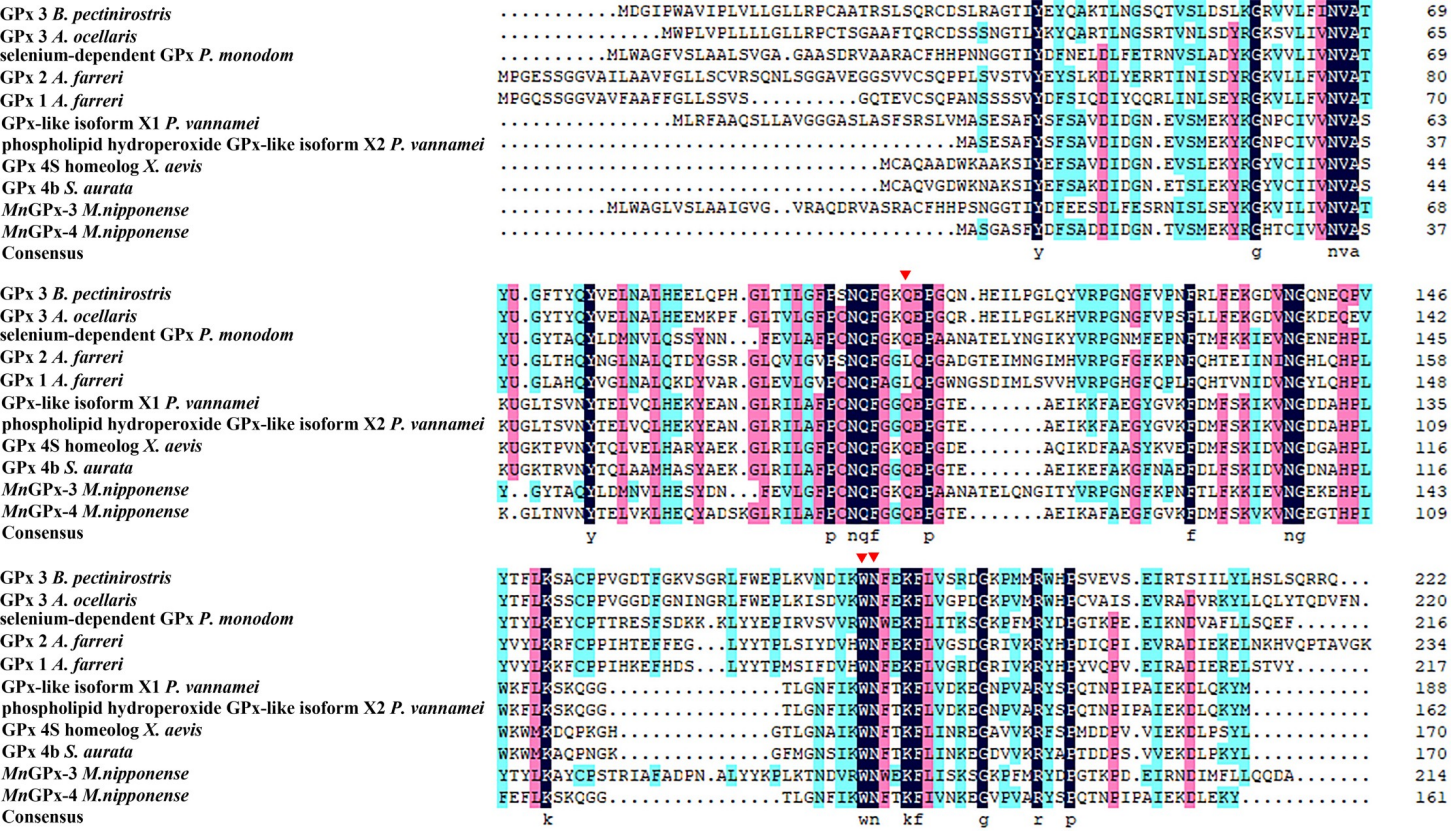
Multiple sequence alignment of the deduced amino acid sequences of *Macrobrachium nipponense* GPXs with those of other known GPXs. GPx 3 *B*. *pectinirostris* (GenBank XP_020782872.1), GPx 3 *A*. *ocellaris* (GenBank XP_023139842.1), selenium-dependent GPx *P*. *monodom* (GenBank AQW41378.1), GPx 1 *A*. *farreri* (GenBank ACF25900.1), GPx 2 *A*. *farreri* (GenBank ACF25901.1), GPx-like isoform X1 *P*. *vannamei* (GenBank XP_027206898.1), phospholipid hydroperoxide GPx-like isoform X2 *P*. *vannamei* (GenBank XP_027206907.1), GPx 4S homeolog *X*. *laevis* (GenBank NP_001165213.1), GPx 4b *S*. *aurata* (GenBank AFY97792.1), *MnGPx-3 M*.*nipponense* (GenBank MK905698), *MnGPx-4 M*.*nipponense* (GenBank MK905699). Red rectangles indicate active sites.

The full-length *MnGPx-3* gene includes an ORF of 648 bp, encoding a 215 amino acid polypeptide with three active sites (Gln102, Trp177 and Asn178). The estimated molecular mass and theoretical pI of this protein were 24.35 kDa and 6.43, respectively. The 5’-untranslated region (UTR) and the 3’-UTR were 99 bp long and 596 bp long, respectively. A tail signal (AATAA) and polyA were also discovered (Fig 1A). *MnGPx-4* comprises an ORF of 492 bp, encoding a 164 amino acid polypeptide with three active sites (Gln75, Trp128 and Asn 129). The deduced protein has an estimated molecular mass of 17.82 kDa and a theoretical pI of 6.30. The 5’-UTR is 259 bp long, and the 3’-UTR is 764 bp long, including a tail signal (AATAA) (Fig 1B). Three potential O-GlcNAc sites were found in *MnGPx-3* (Ser23, Thr79 and Thr198) and *MnGPx-4* (Ser3, Thr155 and Thr149), respectively. Three glycosylation sites in *MnGPx-3* were predicted by the Net NGlyc 1.0 Server, namely, ^50^NIS^52^, ^107^NAT^109^, and ^127^NFT^129^. Two glycosylation sites in *MnGPx-4* were predicted by the Net NGlyc 1.0 Server, namely, ^45^NYT^47^ and ^129^NFT^131^ (Fig 2). The glycosylation site is where the protein chain connects to the sugar chain. Glycosylation plays an important role in protein folding, transport and half-life, as well as being involved in intercellular interaction and antigenicity. Table 1 lists all the primers we used.

### Comparison and phylogenetic analysis of *M*. *nipponense* GPx

**Fig 3:**
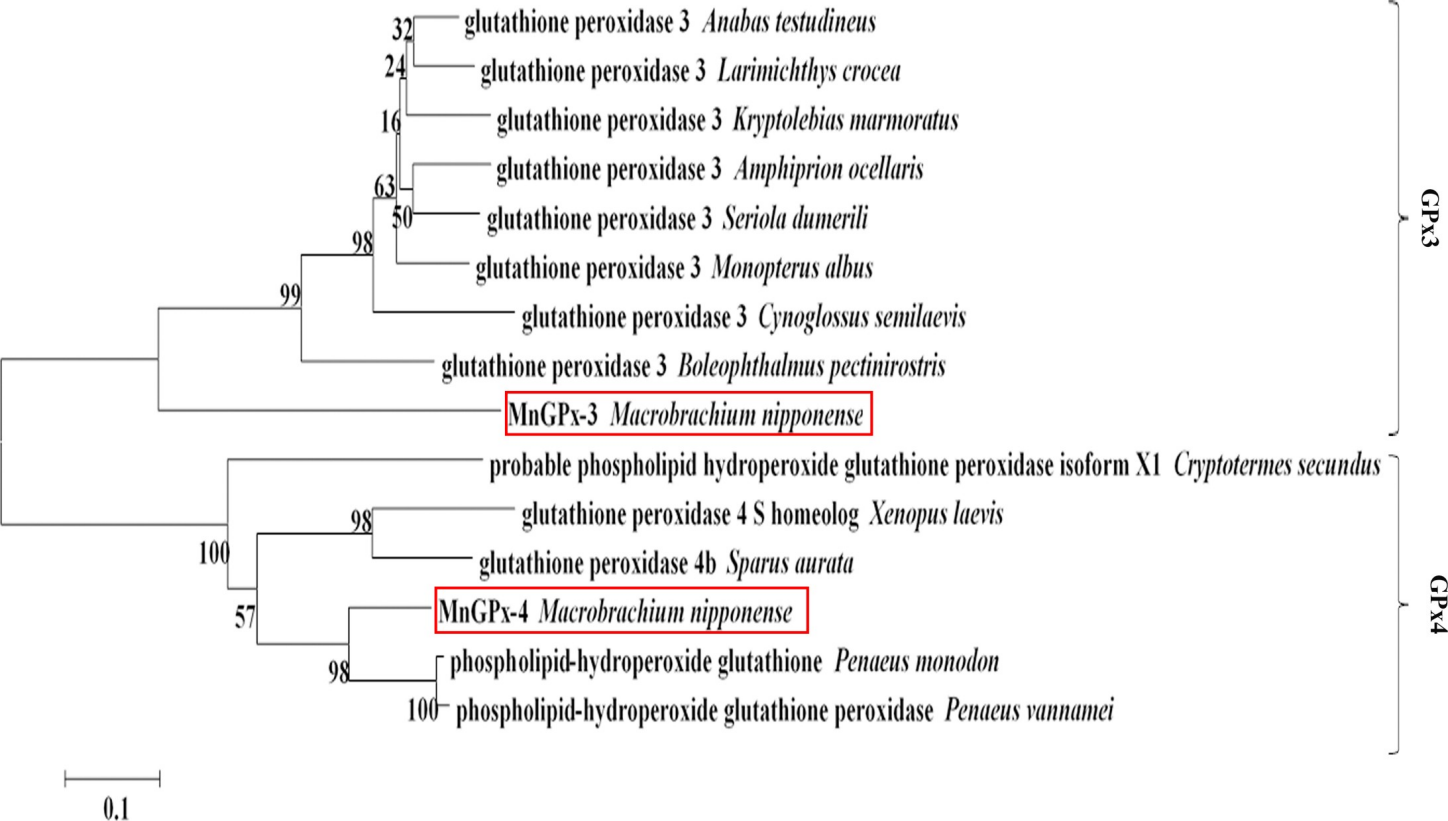
Phylogenetic tree based on GPx sequences generated using the neighbor-joining method in the MEGA 5.10 program with 1000 bootstrap replicates.

BLAST indicates that *MnGPx-3* was 77.57% similar to *P*. *monodon* (GeneBank ID: AQW41378.1) and *MnGPx-4* was 69.84% similar to *P*. *vannamei* (GeneBank ID: XP_027206898.1). Phylogenetic analysis of GPxs from *M*. *nipponense* confirmed that *MnGPx-3* belongs to plasma GPx (GPx-3), while *MnGPx-4* belongs to phospholipid hydroperoxide GPx (GPx-4). As the first GPxs reported on *M*. *nipponense*, they were named *MnGPx-3* and *MnGPx-4*. Most of the deduced amino acid residues in the cDNAs were variable. Phylogenetic tree analysis showed that *MnGPx-3* is most closely related to a GPx in *P*. *monodon*, while *MnGPx-4* is the most closely related to phospholipid hydroperoxide GPx (Fig 3). GenBank accession numbers are listed in Table 2.

**Table 2:** Mature peptide sequences of glutathione peroxidase (GPx) family members.

Species	Gene name	GenBank accession number
*Amphiprion ocellaris*	glutathione peroxidase 3	XP_023139842.1
*Anabas testudineus*	glutathione peroxidase 3	XP_026213175.1
*Boleophthalmus pectinirostris*	glutathione peroxidase 3	XP_020782872.1
*Cryptotermes secundus*	probable phospholipid hydroperoxide glutathione peroxidase isoform X1	XP_023705208.1
*Cynoglossus semilaevis*	glutathione peroxidase 3	XP_008325660.2
*Kryptolebias marmoratus*	glutathione peroxidase 3	XP_017282173.1
*Larimichthys crocea*	glutathione peroxidase 3	XP_010735783.2
*Macrobrachium nipponense*	*MnGPx-3*	MK905698
*Macrobrachium nipponense*	*MnGPx-4*	MK905699
*Monopterus albus*	glutathione peroxidase 3	XP_020449011.1
*Penaeus monodon*	phospholipid-hydroperoxide glutathione	APM86331.1
*Penaeus vannamei*	phospholipid-hydroperoxide glutathione peroxidase peroxidase-like isoform X2	ROT78799.1
*Seriola dumerili*	glutathione peroxidase 3	XP_022607691.1
*Sparus aurata*	glutathione peroxidase 4b	AFY97792.1
*Xenopus laevis*	glutathione peroxidase 4 S homeolog	NP_001165213.1

### Tissue distribution of the two MnGPx subunits

**Fig 4:**
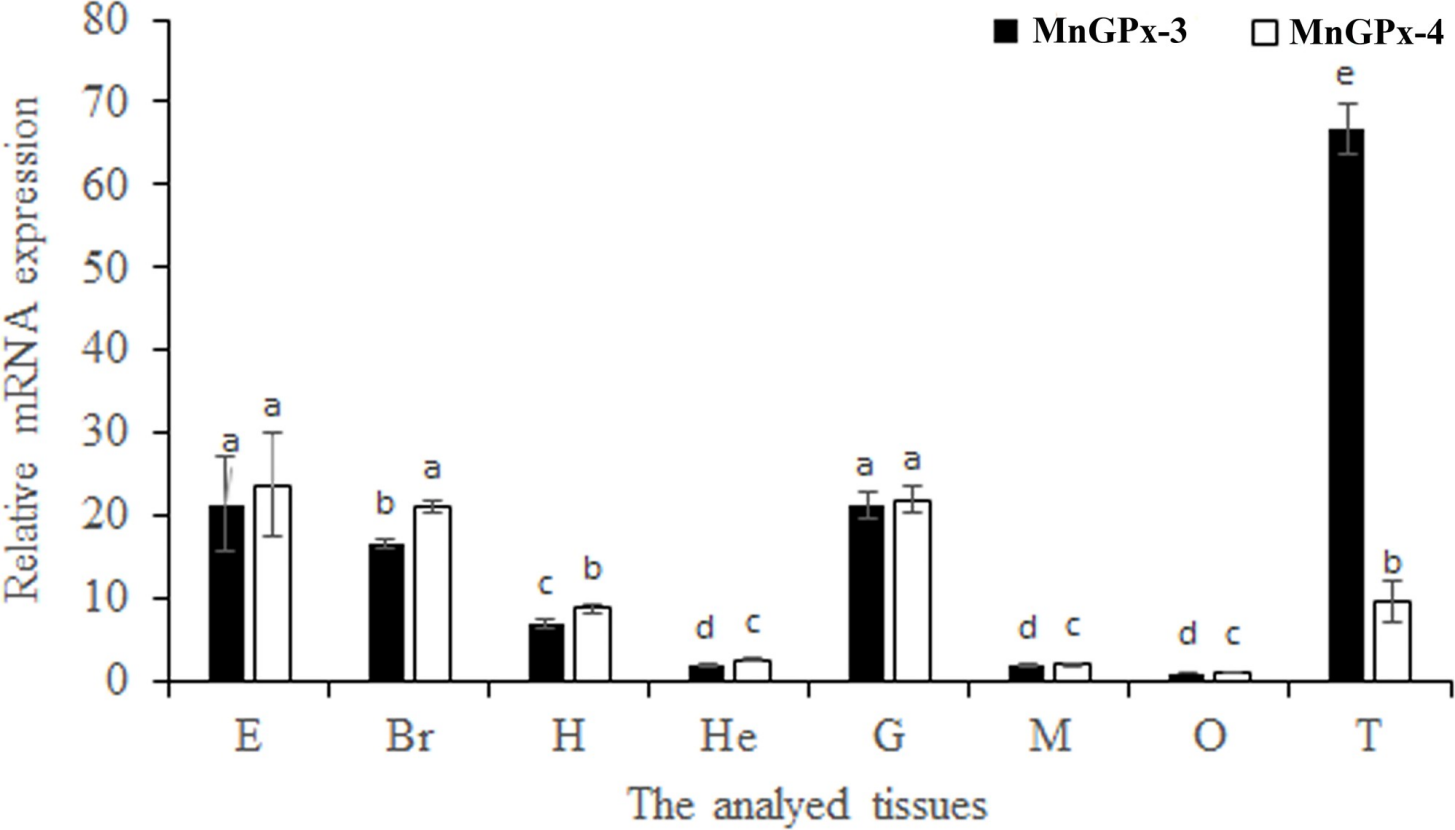
Expression of the two MnGPx subunits in different tissues revealed by QPCR. E, eye; Br, brain; H, heart; He, hepatopancreas; G, gill; M, muscle; O, ovary; T, testis. Data are shown as means ± SD of three replicates in various tissues. Statistical analyses are shown with one-way ANOVA. Different letters indicate a significant difference of the same gene in different tissues (*p* < 0.05).

QPCR (Quantitative real-time reverse transcription PCR) was used to quantify mRNA expression levels of two MnGPxs in different tissue, and both of these two subunits were detected in all tissues tested. *MnGPx-3* mRNA levels were high in the eye, brain, gill and testis. The high-level expression of *MnGPx-4* was similar to *MnGPx-3* mRNA, while *MnGPx-4* had less expression in the testis. The high expression of both *MnGPx-3* and *MnGPx-4* in the gill may indicate that these two subunits play an important role in detoxification and antioxidative damage (Fig 4). Although *MnGPx-3* and *MnGPx-4* are not highly expressed in hepatopancreas, as an important antioxidant tissue, hepatopancreas is still listed as the key research tissue. Based in these expression results, hepatopancreas, gill and muscle tissue were chosen for subsequent experiments.

### Expression of MnGPx subunits in response to changes in environmental oxygen

**Fig 5:**
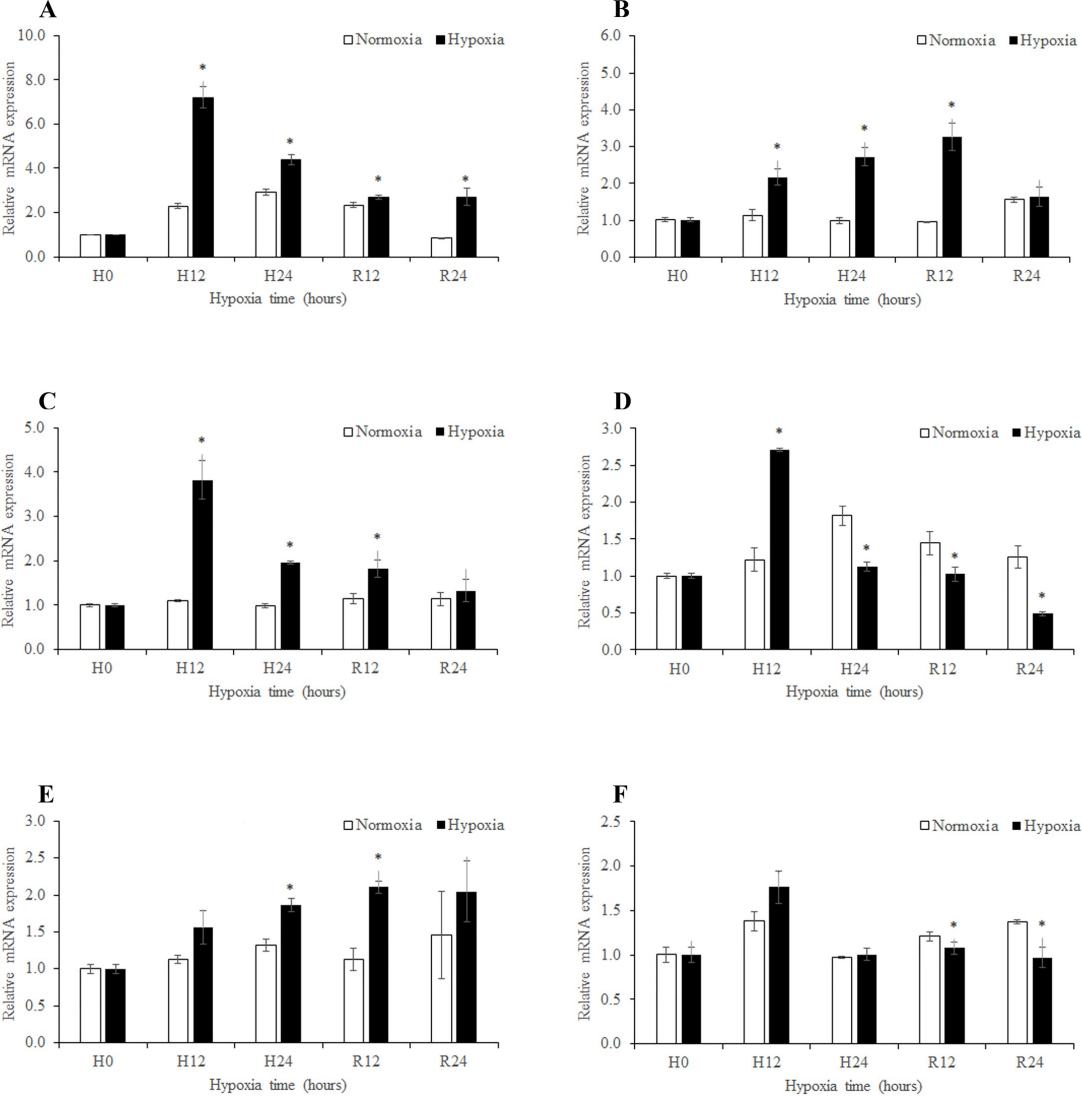
Expression of *MnGPx-3* and *MnGPx-4* in the different tissues at five time points. A, *MnGPx-3* in hepatopancreas; B, *MnGPx-4* in hepatopancreas; C, *MnGPx-3* in gill; D, *MnGPx-4* in gill; E, *MnGPx-3* in muscle; F, *MnGPx-4* in muscle; H0, hypoxia for 0 h; H12, hypoxia for 12 h; H24, hypoxia for 24 h; R12, recovery for 12 h; R24, recovery for 24 h. Data indicated with asterisks are significantly different (p < 0.05) between treatment and control groups. Values are means ± SE for triplicate samples.

The expression patterns of *MnGPx-3* and *MnGPx-4* were determined by qPCR analysis. Following exposure to hypoxia in the hepatopancreas, expression of *MnGPx-3* reached a maximum after 12 h but decreased thereafter. Except at 0 h, the levels of expression were significantly different between the two treatment groups (*p* < 0.05) (Fig 5A). This trend in the expression of *MnGPx-4* reached a peak after reoxygenation for 12 h and then decreased (Fig 5B). Under reoxygenation, *MnGPx-4* expression returned to normal levels, but this was not the case for *MnGPx-3* (Fig 5). Furthermore, the situation of *MnGPx-3* in gill was similar to hepatopancreas, whereas after reoxygenation for 24 h, *MnGPx-3* expression returned to normal levels (*p* < 0.05; Fig 5C). However, *MnGPx-4* expression continued to decrease after reoxygenation (*p* < 0.05; Fig 5D). In muscle, expression of *MnGPx-3* peaked after reoxygenation for 12 h (Fig 5E). The trend in the expression of *MnGPx-4* in muscle is similar to in the gill (Fig 5F).

### ISH of *MnGPx-3* and *MnGPx-4* in hepatopancreas and gill

**Fig 6:**
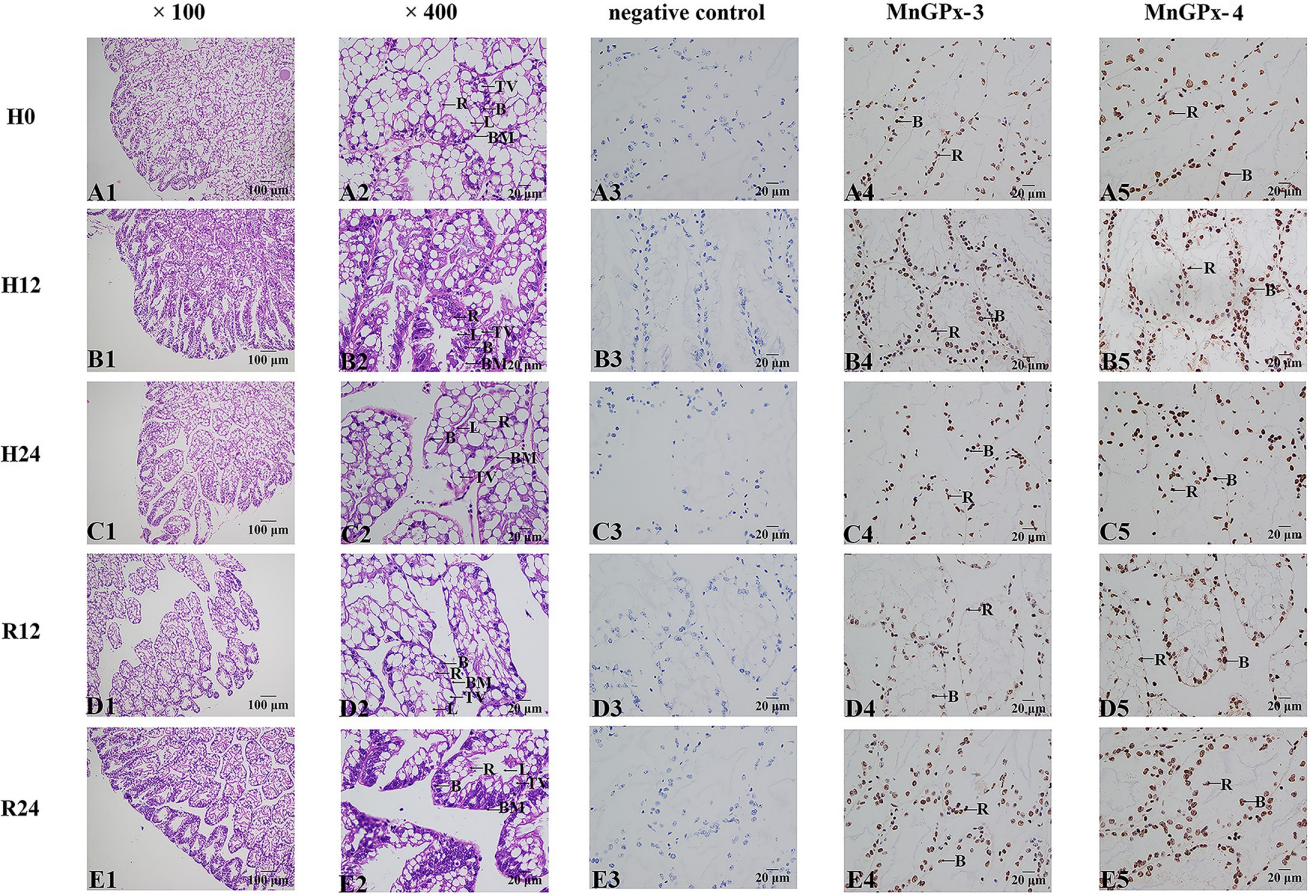
HE staining sections and in situ hybridization of hepatopancreas were performed at five time points. H0, hypoxia for 0 h; H12, hypoxia for 12 h; H24, hypoxia for 24 h; R12, recovery for 12 h; R24, recovery for 24 h; B, secretory cells; BM, basement membrane; L, lumen; R, storage cells; TV, transferred vacuoles.

Hepatopancreas cells usually consist of secretory cells, basement membranes, lumen, storage cells, and transport vesicles. The volume of the transporter vesicle in the hepatopancreas first decreased and then increased during hypoxia. The morphology of the lumen also appeared to have a compression deformation and recovery with hypoxic reoxygenation (Fig 6A2–6E2). ISH analysis indicated that *MnGPx-3* and *MnGPx-4* were located in secretory cells and storage cells. In the negative control experiment, no signal was observed using sense-strand probes (Fig 6A3–6E3).

**Fig 7:**
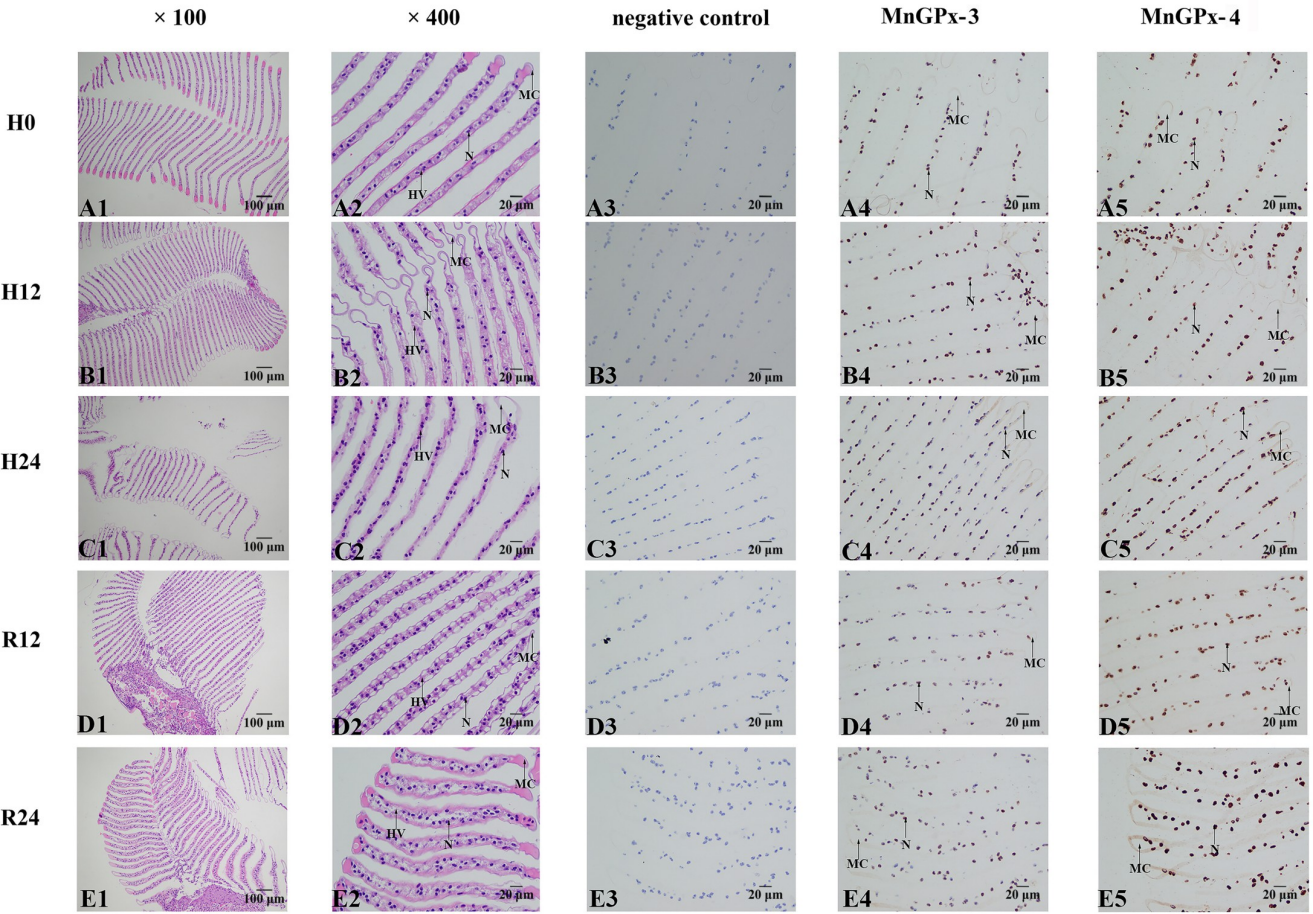
HE staining sections and in situ hybridization of gill were performed at five time points. H0, hypoxia for 0 h; H12, hypoxia for 12 h; H24, hypoxia for 24 h; R12, recovery for 12 h; R24, recovery for 24 h; MC, marginal channel; N, epithelial cell nuclei; HV, hemolymph vessel.

Gill cells are mainly composed of a marginal channel, epithelial cell nuclei and hemolymph vessels. From the results of the section, it was obvious that the marginal channel of the gill cells would be significantly enlarged with an increase in the length of hypoxia, and blood cells flowed into the gill (Fig 7A2–7E2). But as the oxygen returned, the enlarged edge channel gradually returns. After reoxygenation for 24 h, some enlarged marginal channels were not restored to normal levels. ISH analysis showed that *MnGPx-3* and *MnGPx-4* were located in hemolymphatic vessels. In the negative control experiment, no signal was observed with the sense probe (Fig 7A3–7E3).

## Discussion

The cloning and expression of two GPx subunits from *M*. *nipponense* in response to hypoxia and reoxygenation have been reported here. Previous research has shown that the catalytic center of GPx was first identified as composed of Cys or Sec, Gln and Trp, and later was found to be a tetrad with an additional Asn [31–32]. The ternary catalytic center composed of Gln, Trp and Asn indicates that the GPx family has another catalytic mode, which is different from the traditional ternary catalysis and quaternary catalysis. This catalytic method participates in the fixation of selenium by using the stop codon to connect amino acid residues to form a catalytic structure [20]. Furthermore, both *MnGPx-3* and *MnGPx-4* include one Ser and two Thr. In addition, marker codons appeared in the amino acid sequences of both subunits, which could translate the upcoming termination codon into U [18, 33–34]. This indicates that the terminators in the two amino acid sequences are also likely to be translated as U. *MnGPx-3* and *MnGPx-4* are believed to belong to the plasma GPx (GPx-3) class and phospholipid hydroperoxide GPx (GPx-4) class respectively by functional domain analysis and were named according to the established GPx nomenclature system. So far, all available GPxs have been divided into eight different classes based on sequence homology and molecular characteristics, and some are species-specific [19]. *MnGPx-3* was classified as the GPx3 class and *MnGPx-4* was classified as the GPx4 class on the phylogenetic tree. A recent phylogenetic analysis indicated that the arthropod GPx homologues characterized to date form a clade with mammalian GPx3 [35], which is extracellular with broad substrate specificity [36]. A previous study suggested that GPx3 and p53 have a synergistic effect in regulating cellular stress in *P*. *monodon* [20].

In this study, expression of *MnGPx-3* increased at first and then decreased in the gill and hepatopancreas in response to hypoxia, while a slow decline occurred under reoxygenation (Fig 5A and 5C). Multiple studies have suggested that GPx mRNA levels can be an appropriate indicator of oxidative stress which is caused by various xenobiotics [37–38]. *MnGPx-3* mRNA levels rose in the early stage of hypoxia, indicating that the hepatopancreas and gills of *M*. *nipponense* have a strong oxidative stress reaction in response to hypoxia. Decreased expression during hypoxia suggests that animals choose to downregulate metabolism to increase survival when the hypoxic environment does not improve. As previously reported, expression levels of mRNA for crustacean cardioactive peptide in *M*. *nipponense* have the same trend in eyestalk tissue under hypoxia [39]. However, the expression of *MnGPx-3* in muscle showed a continuous upward trend, indicating that the physiological limit of muscles has not been reached after 24 hours of hypoxia. This phenomenon also suggests that muscles respond to hypoxia is different in comparison to the gills and hepatopancreas. Expression of *MnGPx-3* was higher after reoxygenation compared with the control group (Fig 5A, 5C and 5E), indicating that gene expression may be stimulated by a changing level of oxygen in the environment. Similar conclusions have been obtained in the study on hypoxia of glutathione S-transferase in *M*. *nipponense* [1]. The expression of *MnGPx-4* in the hepatopancreas continued to increase during hypoxia and then decreased to normal levels after reoxygenation (Fig 5B). Compared with *MnGPx-3*, the increased expression of *MnGPx-4* was relatively delayed, indicating that *MnGPx-4* had a relatively late response to hypoxia in hepatopancreas. However, *MnGPx-4* mRNA level has the same trend as *MnGPx-3* in the gill (Fig 5D). These results suggest that *MnGPx-4* plays different roles in hepatopancreas and gill. Compared with the control group, the sustained decline of *MnGPx-4* indicates irreversible damage to organs in the gills following hypoxia [4, 40–44]. However, *MnGPx-3* mRNA levels return to normal after reoxygenation. These results suggest that two subunits may be located on different organelles in the gill. This conclusion deserves further study. The expression of *MnGPx-4* follow a different trend to *MnGPx-3* (Fig 5), indicating that *MnGPx-3* and *MnGPx-4* have different functions in the same organization.

In this study, HE staining was used to observe the hepatopancreas and gill sections of *M*. *nipponense*, and ISH was used to locate the two subunits. The volume of the transporter vesicle in the hepatopancreas first decreased and then increased during hypoxia. Previous experiments have demonstrated that the volume of intracellular transport vesicles increased in *Procambarus clarkii* under low pH stress [45], the volume of transport vesicles increased in *Portunus trituberculatus* under low salt environment [46], and the volume and number of transport vesicles increased in *Eriocheir sinensis* nitrogen stress [47], which are consistent with our experimental results. The trend of fluorescence quantification of *MnGPx-3* and *MnGPx-4* first increased and then decreased under hypoxia, and then increased after reoxygenation (Fig 5). Since *MnGPx-3* and *MnGPx-4* were located on secretory cells, we inferred that hypoxia and acute reoxygenation led to an increase in secretory cells. However, this inference will be confirmed by more detailed experiments. These results suggest that the GPx gene is formed in secretory cells and released into cells for a specific function. *MnGPx-3* and *MnGPx-4* were also located in the storage cells by ISH experiments. This suggests that in response to hypoxic stress, GPx genes are formed in secretory cells as well as in storage cells to prepare for subsequent hypoxic stress. In hypoxic conditions, the marginal channels in the gill tissue are enlarged and accompanied by inflow of blood cells. This result is consistent with the results of gill structure changes of *Oncorhynchus mykiss* [48] and *Gymnocypris przewalskii* [49] under hypoxic stress. The results also show that with the increased length of hypoxia, the marginal channels of gill tissue began to expand. However, as oxygen was restored, the expanded marginal channels were gradually restored. After reoxygenation for 24 hours, some marginal channels were still not restored. This result suggests that 24 hours of reoxygenation is not enough to recover the harm of hypoxia to the gill tissue. This prediction coincides with the results of qPCR. ISH showed that both gene subtypes were located in hemolymph vessels. This result indicates that *MnGPx-3* and *MnGPx-4* plays a role in gill tissue through hemolymphatic vessels in response to hypoxic stress.

## Conclusion

In conclusion, we cloned the full-length cDNA sequence of *MnGPx-3* and *MnGPx-4* and analyzed their expression patterns in different tissues of *M*. *nipponense*. We also examined their expression profiles in the hepatopancreas, gill and muscle at five time points during hypoxia and reoxygenation. HE staining was used to observe the hepatopancreas and gill sections of *M*. *nipponense*, and ISH was used to locate the two subunits. The experimental results showed that both subtypes responded to hypoxia, but their expression patterns were different in different tissue. This study provides a basis for understanding the oxidative stress response in *M*. *nipponensis* under hypoxia.

## Supporting information

S1 File (ZIP)
