# Supplementary material for: Molecular cloning, expression, and in situ hybridization analysis of MnGPx-3 and MnGPx-4 from oriental river prawn, Macrobrachium nipponense, in response to hypoxia and reoxygenation
Source: PLoS One. 2020 Feb 21;15(2):e0229171. doi: 10.1371/journal.pone.0229171 (PMC7034814; doi:10.1371/journal.pone.0229171)
Supplement: S1 File — (ZIP) [file pone.0229171.s001.zip › supporting informations/GPxs sequences/figure.docx]

**glutathione peroxidase 3 *Anabas testudineus***

**glutathione peroxidase 3 *Larimichthys crocea***

**glutathione peroxidase 3 *Kryptolebias marmoratus***

**glutathione peroxidase 3 *Amphiprion ocellaris***

**glutathione peroxidase 3 *Seriola dumerili***

**glutathione peroxidase 3 *Monopterus albus***

**glutathione peroxidase 3 *Cynoglossus semilaevis***

**glutathione peroxidase 3 *Boleophthalmus pectinirostris***

**MnGPx-3 *Macrobrachium nipponense***

**probable phospholipid hydroperoxide glutathione peroxidase isoform X1 *Cryptotermes secundus***

**glutathione peroxidase 4 S homeolog *Xenopus laevis***

**glutathione peroxidase 4b *Sparus aurata***

**MnGPx-4 *Macrobrachium nipponense***

**phospholipid-hydroperoxide glutathione *Penaeus monodon***

**phospholipid-hydroperoxide glutathione peroxidase *Penaeus vannamei***

**100**

**98**

**98**

**57**

**100**

**99**

**98**

**50**

**32**

**24**

**63**

**16**

**0.1**
